# Itaconate Promotes Cold Adaptation and Myocardial Protection by Enhancing Brown Adipose Tissue Metabolism

**DOI:** 10.3390/metabo16010066

**Published:** 2026-01-12

**Authors:** Zilong Geng, Xing Liu, Xiao Cheng, Shizhan Xu, Jin Zhang, Ao Tan, Shun Song, Shasha Zhang

**Affiliations:** 1 Key Laboratory of Systems Biomedicine, Shanghai Center for Systems Biomedicine, Department of Cardiovascular Surgery, Xin Hua Hospital, Department of Physical Education, School of Medicine, Shanghai Jiao Tong University, Shanghai 200240, China; 2 Basic Medical Research Center, The Second Affiliated Hospital and Yuying Children’s Hospital, Wenzhou Medical University, Wenzhou 325027, China; 3 College of Biological and Chemical Engineering, Jiaxing University, Jiaxing 314001, China

**Keywords:** itaconate, brown adipose tissue, metabolism, myocardial injury

## Abstract

**Background/Objectives:** Itaconic acid (ITA) is an immunometabolite with anti-inflammatory and metabolic regulatory functions, but its cellular source and role in brown adipose tissue (BAT) remain unclear. This study aims to reveal the expression patterns of the key ITA synthesis gene Irg1 in BAT at different developmental stages and to investigate the effects of cold exposure and exogenous ITA on BAT metabolic function and cardioprotection. **Methods:** Single-cell RNA sequencing was used to analyze the gene expression profiles of stromal vascular fraction (SVF) cells in BAT from P7 neonatal and adult mice. Bioinformatic methods were applied to identify cell types expressing Irg1. Cold exposure (4 °C) and exogenous ITA treatment were employed to evaluate BAT morphology, and the ITA content in BAT was detected using gas chromatography–triple quadrupole mass spectrometry, UCP1 protein expression, and body temperature changes. A transverse aortic constriction (TAC) surgery model was established to induce cardiac dysfunction, and BAT excision was performed to explore the BAT-dependent effects of ITA on myocardial hypertrophy, fibrosis, and cardiac function. **Results:** In P7 neonatal mouse BAT, *Irg1* was predominantly expressed in a subset of interferon-responsive activated macrophages (macrophage27), while in adult mice, it was mainly expressed in neutrophils and a functionally similar macrophage subset (macrophage25). Cold exposure significantly suppressed *Irg1* expression in neutrophils but did not affect its expression in macrophages, also resulting in a significant decrease in ITA content in BAT. Exogenous ITA significantly enhanced BAT thermogenesis under cold conditions, which manifested as reduced lipid droplets, upregulated UCP1 expression, and increased body temperature. In the TAC model, ITA treatment markedly improved cardiac function, attenuated myocardial hypertrophy and fibrosis, and these protective effects were significantly diminished after BAT excision. **Conclusions:** ITA promotes cold adaptation and ameliorates cardiac injury by enhancing BAT metabolic function, and its effects depend on the presence of BAT. This study provides new insights for the treatment of metabolic cardiovascular diseases.

## 1. Introduction

Brown adipose tissue (BAT) is a thermogenic organ unique to mammals. It plays a key role in maintaining body temperature homeostasis and combating metabolic diseases through mitochondrial uncoupling protein 1 (UCP1)-mediated non-shivering thermogenesis [1]. Studies have shown that individuals with active brown fat exhibit a lower prevalence of cardiometabolic diseases, and its presence is independently associated with reduced risks of type 2 diabetes, dyslipidemia, coronary artery disease, cerebrovascular disease, congestive heart failure, and hypertension [2]. Given the protective role of BAT in cardiometabolic health [3,4,5], brown fat and its secreted batokines have emerged as promising therapeutic targets or diagnostic tools for cardiovascular diseases.

The conventional view holds that BAT thermogenesis is primarily regulated by the sympathetic–adrenal axis [6]. However, growing evidence suggests that immune–metabolic crosstalk is also indispensable for modulating BAT activity [7]. Particularly under metabolic stress conditions such as cold exposure or high-fat diet, immune cells residing in BAT can directly reshape the energy metabolism of adipocytes by secreting cytokines or metabolites [8].

Itaconate (ITA) is a mitochondrial metabolite produced by immune-responsive gene 1 (IRG1) through the decarboxylation of cis-aconitate. It was first identified in lipopolysaccharide (LPS)-activated macrophages [9]. ITA not only inhibits succinate dehydrogenase (SDH) to disrupt the inflammatory succinate–mitochondrial ROS–HIF-1α positive feedback loop but also exhibits multiple immunoregulatory functions such as antibacterial, antiviral, and antioxidant effects [10,11]. Recent studies have demonstrated that ITA, as an endogenous metabolite, can alleviate high-fat diet (HFD)-induced obesity by activating BAT-mediated thermogenesis, enhancing fatty acid β-oxidation, and suppressing de novo lipogenesis in BAT, while improving glucose tolerance and dyslipidemia through leptin-independent mechanisms [12]. However, whether ITA is derived from BAT under physiological (non-infectious) conditions and whether it participates in BAT thermogenesis and remote organ protection remain poorly understood.

This study employs single-cell RNA sequencing, cold stimulation, transverse aortic constriction (TAC) models, and BAT excision models to investigate whether BAT possesses endogenous ITA synthesis capacity and its main cellular sources, the regulatory effects of cold exposure on Irg1 expression and ITA synthesis in BAT, the impact of ITA on BAT thermogenesis and its underlying mechanisms, whether ITA exerts protective effects against myocardial injury by enhancing BAT metabolic function.

By integrating single-cell transcriptomics, gene expression regulation, molecular functional experiments, and disease model validation, this research aims to elucidate the immunometabolic role of ITA in BAT from multiple dimensions. It provides new insights into the functions of metabolites in energy homeostasis and cardiovascular health and offers potential targets for immunometabolic therapy in diseases such as obesity, metabolic syndrome, and cardiac dysfunction.

## 2. Materials and Methods

### 2.1. Mice

C57BL/6J mice were used in this study. All animal procedures were conducted in accordance with protocols approved by the Institutional Animal Care and Use Committee (IACUC) of Shanghai Jiao Tong University (Protocol No. A2023206-001). The animals were maintained under standard housing conditions with a 12 h light/dark cycle and provided free access to water and a standard chow diet.

### 2.2. Single Cell RAN Sequencing (scRNA-Seq)

P7 neonatal mice were housed at room temperature with their mothers, who had free access to water and food. The mouse strain was C57BL/6J. Interscapular brown adipose tissue (BAT) was dissected and subjected to enzymatic digestion to isolate cells. The minced tissue was incubated for 45 min at 37 °C under gentle agitation in a digestion cocktail prepared in Hanks’ Balanced Salt Solution (HBSS; Corning^®^, New York, NY, USA) with calcium and magnesium. The enzymatic mixture consisted of 1.5 mg/mL type 1 Collagenase (Merck, Darmstadt, Germany), 2.5 U/mL Dispase II (Merck), and 2% fatty acid-poor bovine serum albumin (BSA; Gibco, Waltham, MA, USA). Following digestion, the dissociated tissue was centrifuged at 500× *g* for 10 min at 4 °C. The adipocyte-containing supernatant was aspirated, and the resulting stromal vascular fraction (SVF) pellet was resuspended in DMEM with 10% FBS (Gibco). This suspension was passed through a 100 µm cell strainer and centrifuged. Erythrocyte contamination was eliminated by resuspending the pellet in 2 mL of sterile ACK lysis buffer, followed by a 5 min incubation on ice. The lysate was filtered through a 40 µm strainer, washed with DMEM/10% FBS, and centrifuged. The final cell pellet was resuspended in PBS with 1.5% BSA. An assessment of suspension quality via microscopy and cell counting demonstrated a cell viability of over 85%. Single-cell RNA-seq libraries were constructed according to the manufacturer’s protocol for Chromium Single Cell 3′ Reagent Kits (10× Genomics, Pleasanton, CA, USA), and sequencing analysis was performed using the 10− Illumina platform.

### 2.3. In Vivo Administration of ITA

Itaconate (Cat# I29204, Sigma-Aldrich, Darmstadt, Germany) was prepared following a previously described method [12]. Briefly, the compound was dissolved in sterile water and filtered through a 0.45 μm membrane for sterilization. Mice subjected to cold exposure or transverse aortic constriction (TAC), along with their corresponding control groups, received daily intragastric administration of either sterile water or itaconate solution at a dose of 300 mg/kg. The duration of treatment was determined based on the cold exposure or TAC modeling protocols.

### 2.4. Surgical Removal of Brown Adipose Tissue (BAT)

6-week-old mice were fasted for 12 h and randomly assigned to either BAT excision or sham surgery groups. Under isoflurane anesthesia, the interscapular region was shaved and disinfected with iodine. A 1.5 cm incision was made between the scapulae, blood vessels were ligated, and the BAT was carefully excised. The incision was then sutured. Sham-operated mice underwent identical procedures except for BAT removal. TAC surgery was performed four weeks later.

### 2.5. Transverse Aortic Constriction (TAC)

Mice were anesthetized with isoflurane (4% for induction, 1.5–2% for maintenance, R510-22-10, RWD, Shenzhen, China) and placed on a heating pad to maintain body temperature at 37 °C. After endotracheal intubation, mechanical ventilation was initiated (tidal volume: ~200 µL, respiratory rate: 120 breaths/min). A 1 cm longitudinal incision was made between the second and third ribs along the left sternal border. The thoracic cavity was carefully opened to expose the aortic arch. A 28-gauge blunt needle was placed parallel to the aorta, which was then ligated together with the needle using a 4-0 silk suture. The needle was promptly removed to create a standardized constriction. Successful constriction was confirmed by visible pulsation and dilation proximal to the ligation site. Muscle and skin layers were sutured sequentially. Sham-operated mice underwent the same procedure without aortic ligation. Echocardiography was performed four weeks post-surgery to validate the model.

### 2.6. Cold Exposure

Ten-week-old mice were individually housed. The control group was maintained at room temperature (23 °C ± 2 °C), while the experimental group was exposed to 4 °C for 4 days with ad libitum access to food. After euthanasia via CO_2_ asphyxiation, interscapular BAT was collected and either fixed in 4% PFA (SB-C010, Share-bio, Shanghai,China) or snap-frozen in liquid nitrogen or RNAlater (AM7024, Invitrogen, Carlsbad, CA, USA).

### 2.7. Gas Chromatography–Triple Quadrupole Mass Spectrometry (GC/MS)

BAT samples (70 mg) were collected from 10-week-old wild-type mice subjected to the following conditions: 4 °C cold exposure for 2 days, gastric administration of ITA for 4 days (300 mg/kg), and untreated controls. The tissues were homogenized in 100 μL of double-distilled water and subjected to ultrasonic disruption. Subsequently, 200 μL of a methanol:chloroform (3:1) mixture was added. The samples were centrifuged at 12,000 rpm and 4 °C for 10 min, and 200 μL of the supernatant was collected. The liquid was evaporated under vacuum centrifugation, and the remaining solid was mixed with 40 μL of pyridine and 80 μL of BSTFA. The mixture was reacted at 70 °C for 1.5 h and then analyzed by GC/MS. Additionally, ITA powder (Sigma-Aldrich, Darmstadt, Germany,Catalog No. I29204) was used to prepare standard solutions at concentrations of 0.1 μg/mL, 1 μg/mL, and 10 μg/mL.

### 2.8. Wheat Germ Agglutinin (WGA) Staining

Paraffin sections were dewaxed and rehydrated through an ethanol gradient. Antigen retrieval was performed in sodium citrate buffer (pH 6.0) at 95 °C for 20 min. Sections were blocked for 30 min with 5% normal donkey serum and 0.1% Triton X-100 in PBS, then incubated with Alexa Fluor 647-conjugated wheat germ agglutinin (1:200 dilution, W32466, Invitrogen, Carlsbad, CA, USA) for 30 min. Nuclei were counterstained with Hoechst 33,342 (R37605, Thermo Fisher Scientific, Waltham, MA, USA), and slides were mounted with ProLong Gold Antifade Reagent (P36934, Thermo Fisher Scientific, Waltham, MA, USA). Images were acquired using a Nikon A1Si confocal microscope (Nikon Corporation, Tokyo, Japan) and analyzed with ImageJ Version 2.3.0.

### 2.9. RT-qPCR

Total RNA was extracted using an RNAsimple Total RNA Kit (DP419, TIANGEN, Beijing, China) according to the manufacturer’s instructions. cDNA was synthesized from 0.1–1 μg RNA using a Hifair^®^ II First Strand cDNA Synthesis Kit (11119ES60, YEASEN, Shanghai, China). Quantitative PCR was performed with Hieff^®^ qPCR SYBR Green Master Mix (11203ES08, YEASEN, China) on an ABI Prism 7500 system (Applied Biosystems, Waltham, MA, USA). Primer sequences were as follows:

*18S rRNA*: F-GTAACCCGTTGAACCCCATT, R-CCATCCAATCGGTAGTAGCG

*UCP1*: F-CGTCCCCTGCCATTTACTGT, R-GACCCGAGTCGCAGAAAAGA

*IFN-γ*: F-CGGCACAGTCATTGAAAGCC, R-TGCATCCTTTTTCGCCTTGC

### 2.10. Echocardiography

Transthoracic echocardiography was performed using a Visual Sonics 2100 system (FUJIFILM VisualSonics, Toronto, Canada) with an MS-400 (30 MHz) transducer. Mice were anesthetized with 5% isoflurane (R510-22-10, RWD, Shenzhen, China) in oxygen (21% O_2_ + 79% N_2_) and maintained under 1.5% isoflurane. Heart rates were stabilized between 450–500 bpm before imaging. Left ventricular parameters, including ejection fraction (EF), fractional shortening (FS), Left Ventricular Internal Dimension at diastole (LVIDd), and Left Ventricular Internal Dimension at systole (LVIDs), were measured from M-mode images. Operators were blinded to experimental groups. Sample sizes were determined based on pilot studies or established protocols.

### 2.11. Histochemistry

Hearts were fixed in 4% PFA for 24 h at 4 °C, dehydrated through an ethanol series, cleared in xylene, and embedded in paraffin. Sections (6 μm) were stained using Masson’s Trichrome Kit (60532ES66, YEASEN, China) or H&E Kit (60524ES60, YEASEN, China) according to manufacturer instructions.

### 2.12. Statistical Analysis

Data were analyzed using GraphPad Prism (Version 9.0.0) and expressed as mean ± SD. Normality was assessed with the Shapiro–Wilk test. For two-group comparisons, two-tailed unpaired *t*-tests or Mann–Whitney U tests were used as appropriate. Multiple comparisons were analyzed by one-way ANOVA with Tukey’s post hoc test or Kruskal–Wallis test. A *p*-value < 0.05 was considered statistically significant.

## 3. Results

### 3.1. Analysis of Irg1 Expression in Neonatal Mouse Brown Adipose Tissue

**Figure 1:**
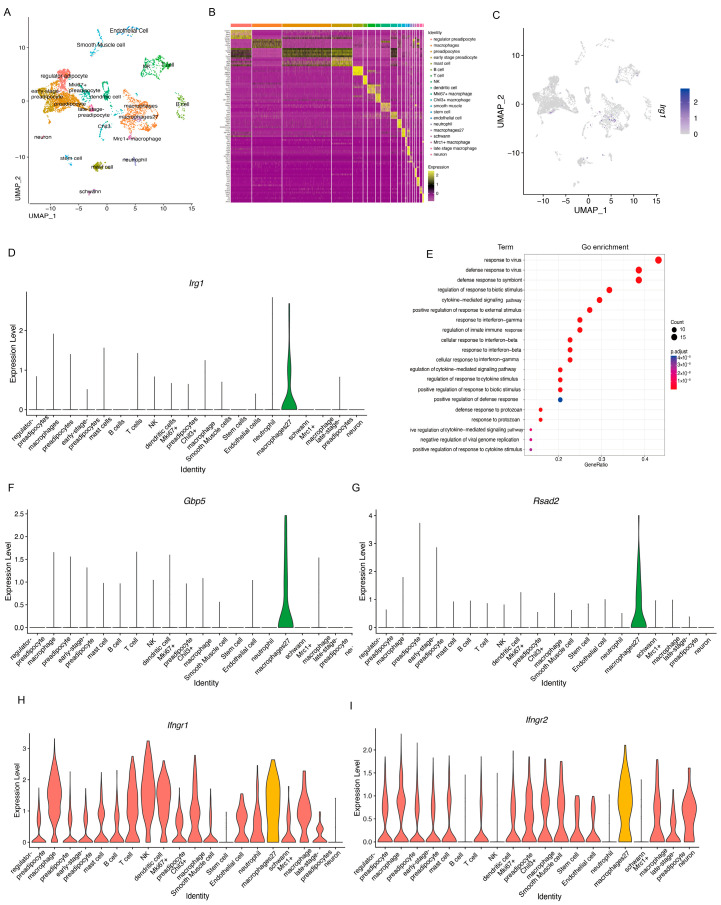
Single-cell RNA sequencing analysis of iBAT SVF revealed a macrophage cell cluster with high Irg1 expression and IFNγ-induced activation. (**A**) t-distributed stochastic neighbor embedding (tSNE) plot of P7 iBAT SVF (*n* = 6). (**B**) Heatmap of differentially expressed genes for each cell cluster. (**C**) UMAP projection of single-cell transcriptomes from SVF cells based on gene expression clustering. FeaturePlots showed Irg1 expression in SVF cells. (**D**) Violin plot displaying the distribution of *Irg1* expression. (**E**) Gene Ontology (GO) analysis of highly expressed genes in macrophage27. (**F**) *Gbp5* expression visualized by ViolinPlots. (**G**) *Rsad2* expression visualized by ViolinPlots. (**H**) *Ifngr1* expression visualized by ViolinPlots. (**I**) *Ifngr2* expression visualized by ViolinPlots.

To determine whether brown adipose tissue (BAT) possesses the capacity to synthesize endogenous Itaconate (ITA), interscapular BAT (iBAT) was harvested from thermogenically active wild-type P7 neonatal mice [13]. Stromal vascular fraction (SVF) cells were isolated and subjected to single-cell RNA sequencing. Unsupervised dimensionality reduction via t-SNE was performed on scRNA-seq data from 5720 SVF cells obtained from six healthy wild-type P7 mice, identifying 42 transcriptionally distinct pre-clustered groups. Based on cell-specific markers and significantly enriched genes, 20 cell populations were annotated (Figure 1A), mainly including adipocyte stem cells, preadipocytes, macrophages, T cells, B cells, mast cells, dendritic cells, neutrophils, smooth muscle cells, endothelial cells, and neural cells (Figure 1B). Notably, the key ITA synthesis gene *Irg1* was predominantly enriched in a specific macrophage subset—macrophage27 (Figure 1C,D). Further molecular characterization revealed that macrophage27 highly expressed genes involved in interferon response pathways (Figure 1E). Additionally, this subset specifically expressed *Gbp5* (Figure 1F) and *Rsad2* (Figure 1G), established markers of IFN-γ-induced classically activated macrophages [14,15]. Violin plots confirmed high expression of IFN-γ receptors *Ifngr1* and *Ifngr2* in macrophage27 (Figure 1H,I). These findings indicate that macrophage27 represents a population of IFN-γ-induced activated macrophages in BAT capable of expressing *Irg1*, consistent with reported TLR- or IFN-γ-dependent induction of IRG1 in myeloid cells [6,16]. Thus, macrophage27 is a likely cellular source of ITA in iBAT.

### 3.2. Irg1 Expression in Adult Mouse BAT and the Effects of Cold Exposure

**Figure 2:**
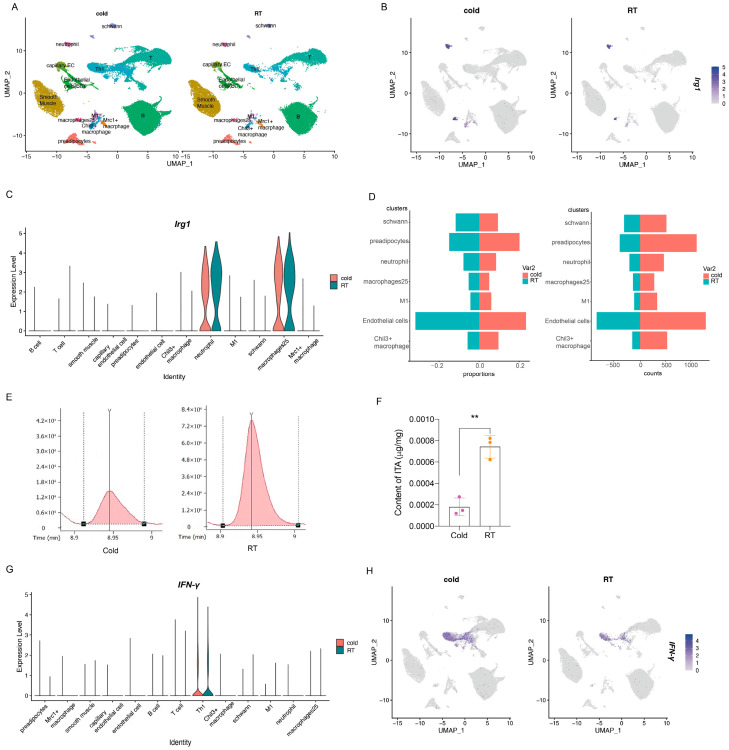
Single-cell RNA sequencing analysis of *Irg1* and *IFN-γ* at cellular and transcriptional levels in iBAT SVF after 2-day cold stimulation and room temperature treatment. (**A**) t-distributed stochastic neighbor embedding (tSNE) plot of iBAT SVF from 2-day cold-stimulated and room temperature control groups. (**B**) FeaturePlots showing the expression of *Irg1* in SVF cells. (**C**) Violin plot displaying the expression of *Irg1* (*p* value = 0.5871033 for macrophage25 and *p* value = 0.00092 for neutrophil, Wilcox test). (**D**) Statistics of various cell types under cold stimulation and room temperature conditions. (**E**) Chromatographic peaks of ITA in BAT under room temperature and cold stimulation. (**F**) ITA content in BAT under room temperature and cold stimulation. (**G**) Violin plot showing the expression of *IFN-γ*, with no significant change in average expression in TH1 (*p* value = 0.6363153, Wilcox test). (**H**) FeaturePlots showing the expression of *IFN-γ* in SVF cells (** *p* < 0.01).

Given that thermogenic activity in adult mouse BAT is markedly reduced compared to P7 neonates, we investigated whether *Irg1* is expressed in adult BAT and how cold exposure affects its expression. We analyzed single-cell transcriptomic data of SVF cells from adult BAT under room temperature (20,464 cells), 2-day cold exposure (30,564 cells), and 7-day cold exposure (19,091 cells) obtained from the GEO database. Thirteen cell populations were identified based on marker expression and gene enrichment (Figure 2A). In adult BAT, *Irg1* was primarily expressed in neutrophils and macrophage25 (Figure 2B). Macrophage25 also expressed *Gbp5* (Supplementary Figure S1A), *Rsad2* (Supplementary Figure S1B), *Ifngr1* (Supplementary Figure S1C), and *Ifngr2* (Supplementary Figure S1D). Gene ontology analysis indicated functional similarity between neonatal macrophage27 and adult macrophage25 (Figure 1E, Supplementary Figure S1E). Under cold stimulation, *Irg1* expression in macrophage25 remained unchanged (*p* = 0.587) (Figure 2C), and the abundance of macrophage25 increased (Figure 2D, Supplementary Figure S1F). In contrast, cold exposure significantly suppressed *Irg1* expression in neutrophils (*p* = 0.00092) (Figure 2C, Supplementary Figure S1G) and increased neutrophil numbers (Figure 2D, Supplementary Figure S1F). To directly determine the impact of cold stimulation on ITA synthesis, we employed GC/MS and found that cold exposure indeed led to a significant decrease in ITA content within BAT (Figure 2E,F). After two days of cold exposure, the ITA level in BAT was only 18.9% of that in the room temperature group. These results suggest that macrophage-derived ITA expression may be cold-resistant to maintain basal functions, whereas neutrophils modulate ITA expression in response to environmental stimuli.

To examine whether reduced *Irg1* expression in neutrophils was due to attenuated inflammation in cold-exposed BAT [17], we analyzed the expression of IFN-γ, a key transcriptional inducer of *Irg1*. The proportion of TH1 cells—the primary IFN-γ-secreting population—increased after 2 and 7 days of cold exposure (Supplementary Figure S1F,H,I). Although mean *IFN-γ* expression per TH1 cell did not change significantly (Figure 2G), An increase in TH1 proportion, coupled with no significant change in *IFN-γ* expression per cell, suggests a potential rise in total *IFN-γ* production (Figure 2H). Consistent with this, RT-qPCR analysis of SVF RNA revealed significantly higher *IFN-γ* expression after 2 days of cold exposure compared to the room temperature group (Supplementary Figure S1J). These data indicate that IFN-γ is not the sole determinant of *Irg1* induction.

### 3.3. ITA Enhances BAT Thermogenesis Under Cold Stimulation

**Figure 3:**
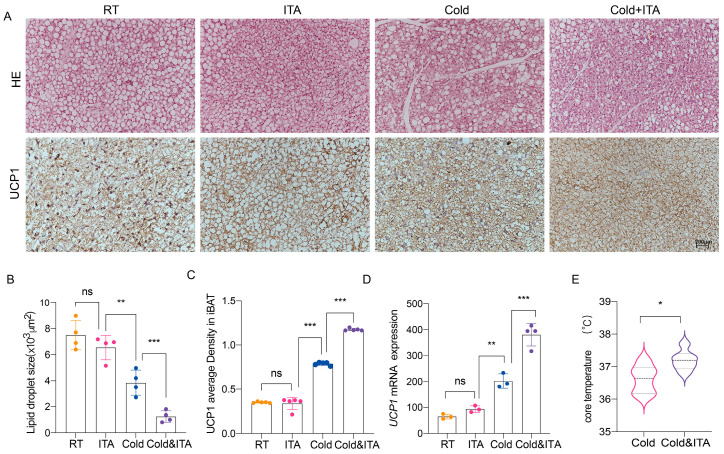
ITA can significantly enhance the thermogenic capacity of BAT under cold stimulation. (**A**) HE staining and UCP1 immunohistochemical staining of BAT under room temperature (RT) and cold stimulation conditions treated with ITA (Scale bars = 200 μm). (**B**) Statistical analysis of the average lipid droplet area in BAT of each group based on HE staining (*n* = 4 for each group, 2-tailed Student’s *t*-test). (**C**) Statistical analysis of UCP1 expression in BAT of each group based on UCP immunohistochemical staining results (*n* = 5 for each group, 2-tailed Student’s *t*-test). (**D**) Detection of *UCP1* transcriptional levels in BAT under RT and Cold conditions or treated with ITA (*n* = 3 for each group), Statistical significance was determined by One-way ANOVA post hoc with Tukey multiple comparison test. (**E**) Rectal temperature after 4 days of ITA treatment under cold stimulation conditions (*n* = 6 for each group, 2-tailed Student’s *t*-test) (* *p* < 0.05, ** *p* < 0.01, *** *p* < 0.001, ns: non-significant).

To determine whether decreased *Irg1* expression during cold exposure reflects substrate-limited feedback inhibition or a negative effect on BAT thermogenesis, we evaluated the impact of exogenous ITA on cold-induced thermogenesis. After 4 days of ITA treatment under room temperature (RT), GC/MS analysis showed that intragastrically administered ITA effectively absorbed by the BAT, increasing the ITA content by approximately 82.9-fold (Supplementary Figure S2A,B). Lipid droplets in BAT were slightly smaller than controls, though not significantly (Figure 3A,B). UCP1 expression increased modestly but not significantly (Figure 3A,C). However, after 4 days of cold exposure, ITA-treated mice exhibited significantly smaller lipid droplets (Figure 3A,B), elevated UCP1 expression (Figure 3A,C,D), and increased rectal temperature (Figure 3E) compared to controls. These results indicate that ITA does not affect BAT thermogenesis under physiological conditions but enhances cold-induced thermogenesis, supporting the hypothesis that cold-induced reduction in *Irg1* is primarily due to feedback inhibition resulting from limited substrate availability.

### 3.4. ITA Attenuates Myocardial Injury by Enhancing BAT Metabolism

**Figure 4:**
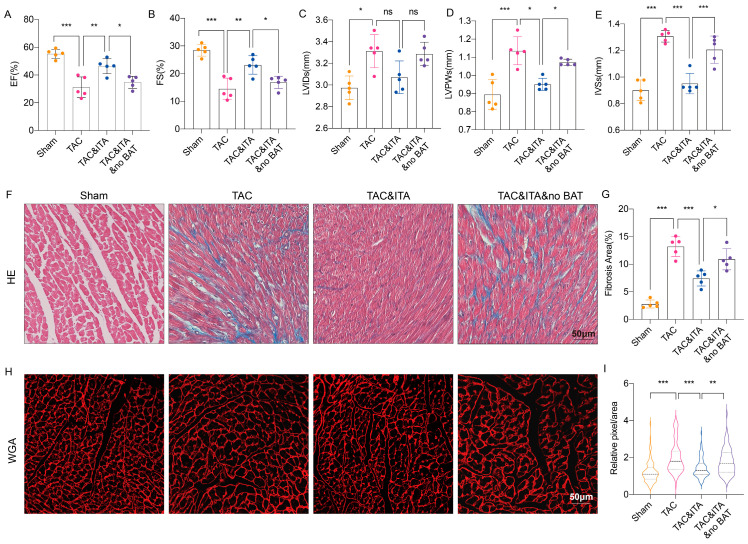
ITA alleviates myocardial injury through BAT. (**A**–**E**) Echocardiogram illustrates impaired EF (**A**), FS (**B**), and increased LVIDs (**C**), LVPWs (**D**), IVSs (**E**) after TAC. Intragastric administration of ITA for two consecutive weeks improved these parameters, but no significant improvement was observed in the BAT-removed group. Sham-operated groups were used as controls (*n* = 5 for each group, Two-tailed Student *t* test). (**F**,**G**) Masson staining. The fibrotic regions (blue) in 6 different fields per heart (5 hearts per group) were measured using ImageJ (2-tailed Student’s *t*-test; scale bar = 50 μm). (**H**,**I**) Cardiomyocyte size illustrated by wheat germ agglutinin staining. Violin plots represent 100 cardiomyocytes from 3 measured hearts (Mann–Whitney test; scale bar = 50 μm). (* *p* < 0.05, ** *p* < 0.01, *** *p* < 0.001, ns: non-significant).

To investigate whether ITA confers protection against pathological cardiac injury via BAT metabolic activation, a transverse aortic constriction (TAC) model was employed. TAC or myocardial infarction (MI) induces hypoxia and apoptosis in BAT, impairing thermogenesis and leading to hypothermia [18]. We combined TAC surgery with BAT excision to study the BAT-dependent cardioprotective effects of ITA. BAT was surgically removed at 6 weeks of age, TAC was performed at 10 weeks, and daily intragastric ITA administration began at 11 weeks. Four weeks post-TAC, mice exhibited impaired cardiac function (Figure 4A,B), left ventricular dilation (Figure 4C), ventricular wall and septal thickening (Figure 4D,E), myocardial fibrosis (Figure 4F,G), and cardiac hypertrophy (Figure 4H,I). ITA treatment significantly improved cardiac function, ameliorated hypertrophy and fibrosis (Figure 4A–I). Critically, the protective effects of ITA were substantially attenuated in BAT-excised mice (Figure 4A–I). These results demonstrate that ITA alleviates myocardial injury by enhancing BAT metabolic function.

## 4. Discussion

This study systematically investigated the cellular sources, regulatory mechanisms, and physiological functions of itaconate (ITA) in brown adipose tissue (BAT) under cold-induced thermogenesis and cardioprotection by integrating single-cell transcriptomics, molecular biology, and animal models. The major innovative findings include: (1) identification of a macrophage subpopulation in BAT that is activated by type II interferon (IFN-γ) and highly expresses Irg1; (2) demonstration that adult BAT retains ITA production primarily from neutrophils and macrophages under basal conditions, while cold exposure reduces Irg1 expression in neutrophils but does not alter Irg1 expression in macrophages; (3) discovery that exogenous ITA further enhances BAT thermogenesis under cold stimulation; (4) elucidation that under pathological conditions, ITA significantly alleviates pressure overload-induced myocardial injury by enhancing BAT metabolic function, an effect that is BAT-dependent.

Initially, we found that in neonatal mouse BAT, Irg1 is predominantly expressed in a subset of interferon-responsive activated macrophages (macrophage27), which highly express interferon response-related genes and receptors. This is consistent with previous reports that Irg1 expression is regulated by the IFN-γ signaling pathway [16]. This finding suggests the existence of an immunometabolically specialized macrophage subset within BAT that may serve as an important local source of ITA. Notably, the expression pattern of Irg1 shifts in adult mouse BAT, where it is mainly expressed in neutrophils and another functionally similar macrophage subset (macrophage25), indicating that the cellular source of ITA may dynamically change with developmental stage and metabolic status. A recent study has reported that neutrophils can be induced to express Irg1 in contexts such as tumors or autoimmune diseases [19]. Our observation that cold stimulation significantly downregulates Irg1 expression in neutrophils suggests that ITA synthesis in neutrophils exhibits environmental plasticity. Additionally, there is currently no definitive evidence in the literature directly demonstrating Irg1 expression in mature adipocytes. Similarly, we did not observe significant expression of Irg1 in preadipocyte subpopulations either.

Cold stimulation experiments revealed a complex regulatory mechanism for ITA synthesis. Although the number of IFN-γ-producing TH1 cells increased after cold exposure, Irg1 expression levels remained stable in macrophages but decreased significantly in neutrophils, and the ITA content in BAT declined. This phenomenon may reflect prioritization in metabolic substrate competition: under cold stress, cis-aconitate is preferentially diverted into the tricarboxylic acid (TCA) cycle to meet the energy demands of thermogenesis, leading to a relative shortage of substrate for ITA synthesis and consequent feedback inhibition of Irg1 expression. This refined regulatory mechanism ensures optimized allocation of energy metabolism during cold adaptation. This aligns closely with the observation by Bambouskova et al. that in macrophages “metabolic reprogramming dictates ITA levels” [20]. Yu et al. demonstrated that itaconate (ITA) markedly alleviates high-fat-diet-induced obesity and improves glucose and lipid metabolism in mice by activating brown adipose tissue (BAT) thermogenesis, and this process is independent of leptin signaling [12]. This prompts us to consider whether exogenous increase in ITA during cold stimulation can promote cold adaptation. Importantly, our study found that exogenous ITA treatment significantly enhanced BAT thermogenic capacity under cold conditions, manifested as reduced lipid droplets, upregulated UCP1 expression, and increased body temperature. This result not only confirms the promotive effect of ITA on BAT thermogenesis but also supports the view that the decrease in endogenous Irg1 expression is primarily due to substrate limitation rather than a detrimental effect of ITA itself on heat production. The administration of exogenous ITA circumvents this negative feedback mechanism, thereby proposing “cold exposure + ITA” as a synergistic interventional strategy.

The most exciting finding pertains to the mechanism of ITA’s cardioprotective effects. Using a TAC model, we demonstrated that ITA significantly improved cardiac function, attenuated myocardial hypertrophy and fibrosis, but this protective effect was markedly diminished in BAT-excised mice, indicating that ITA exerts its cardioprotective role primarily by enhancing BAT metabolic function. TAC-induced myocardial cell pyroptosis is a key direct cause of cardiac function deterioration and remodeling [21]. The cardioprotective effect of ITA after TAC may be related to its inhibition of pyroptosis [22]. The improvement in cardiac function in the ITA-only treatment group may also be attributed to the activation of the Nrf2/ARE pathway [23] and the suppression of inflammatory cell infiltration [24]. Previous research has focused on BAT-secreted factors such as neuregulin-4 (NRG4) [25], batokines including FGF21 [26], and 12,13-diHOME [3] in improving cardiac function. The protective effect of ITA against myocardial injury, mediated through BAT metabolic activation, may be due to an increase in the secretion of these factors following metabolic enhancement, while some of these factors don’t have a direct promoting effect on the thermogenic function of BAT [27,28].

In this study, we found that cold stimulation reduces the transcriptional level of Irg1 in neutrophils. The specific molecular mechanisms underlying both the protein expression and enzymatic activity of Irg1 in brown adipose tissue after cold stimulation, as well as how cold stimulation downregulates Irg1 transcription in neutrophils, warrant further investigation. Additionally, brown adipose tissue can improve cardiac function post-injury by secreting various cytokines. Therefore, whether enhanced brown adipose activity induced by ITA treatment produces cardioprotective cytokines and how these functional protective factors regulate cardiac repair after injury merit further exploration.

## 5. Conclusions

In summary, this study not only reveals the cellular sources and regulatory mechanisms of ITA synthesis in BAT but also demonstrates the dual role of ITA in promoting cold adaptation and cardioprotection through enhancing BAT metabolic function. These findings expand our understanding of the role of immunometabolites in energy homeostasis and cardiovascular health, laying a theoretical foundation for developing ITA-based therapeutic strategies. Future studies are needed to further elucidate the specific molecular mechanisms by which ITA acts on BAT and its potential applications in other metabolic diseases.

## Data Availability

The sequencing data generated in this study are available in the NCBI GEO under accession number GSE160585, and all other relevant data can be obtained from the corresponding author upon reasonable request.

## Supplementary Materials

The following supporting information can be downloaded at: https://www.mdpi.com/article/10.3390/metabo16010066/s1, Supplemental Figure S1. Single-cell RNA sequencing analysis of cellular and transcriptional levels in iBAT SVF under cold stimulation and room temperature treatment. (A) FeaturePlots showing the expression of Gbp5 in BAT SVF cells after 2-day cold stimulation and under room temperature conditions. (B) FeaturePlots showing the expression of Rsad2 in BAT SVF cells after 2-day cold stimulation and under room temperature conditions. (C) FeaturePlots showing the expression of Ifngr1 in BAT SVF cells after 2-day cold stimulation and under room temperature conditions. (D) FeaturePlots showing the expression of Ifngr2 in BAT SVF cells after 2-day cold stimulation and under room temperature conditions. (E) Gene Ontology (GO) analysis of highly expressed genes in macrophage25 after 2-day cold stimulation. (F) Statistics of cell type proportions in BAT SVF after 2-day cold stimulation and under room temperature conditions. (G) A dot plot showing a significant decrease in Irg1 expression in neutrophils after 2-day cold stimulation. (H) t-distributed stochastic neighbor embedding (tSNE) plot of iBAT SVF from 7-day cold-stimulated and room temperature control groups. (I) Statistics of cell type proportions in BAT SVF after 7-day cold stimulation and under room temperature conditions. (J) The transcriptional level of IFN-γ in iBAT adipose tissue of mice under cold stimulation for 7 days and room temperature conditions was detected by RT-qPCR. Statistical significance was determined by 2-tailed Student’s *t*-test (** *p* < 0.01, ns: non-significant). Supplemental Figure S2. Analysis of ITA Content in BAT after Intragastric Administration of ITA. (A) Chromatographic peaks of ITA in BAT from ITA-treated and control groups. (B) ITA content in BAT from ITA-treated and control groups (*** *p* < 0.001).

## References

[1] Cannon B., Nedergaard J. (2004). Brown adipose tissue: Function and physiological significance. Physiol. Rev..

[2] Becher T., Palanisamy S., Kramer D.J., Eljalby M., Marx S.J., Wibmer A.G., Butler S.D., Jiang C.S., Vaughan R., Schöder H. (2021). Brown adipose tissue is associated with cardiometabolic health. Nat. Med..

[3] Badi I., Antoniades C. (2021). Brown Adipose Tissue and the Take (12,13-di)HOME Message to the Heart. Circulation.

[4] Thoonen R., Hindle A.G., Scherrer-Crosbie M. (2016). Brown adipose tissue: The heat is on the heart. Am. J. Physiol. Heart Circ. Physiol..

[5] Xu Z., Li H., Cao G., Li P., Zhou H., Sun Y. (2024). The protective role of brown adipose tissue in cardiac cell damage after myocardial infarction and heart failure. Lipids Health Dis..

[6] Geoghegan G., Simcox J. (2023). SON-light activation of glucose regulation. Cell.

[7] Villarroya F., Cereijo R., Villarroya J., Giralt M. (2017). Brown adipose tissue as a secretory organ. Nat. Rev. Endocrinol..

[8] Bertola A., Gallerand A., Ivanov S. (2022). Immune cell involvement in brown adipose tissue functions. Discov. Immunol..

[9] Michelucci A., Cordes T., Ghelfi J., Pailot A., Reiling N., Goldmann O., Binz T., Wegner A., Tallam A., Rausell A. (2013). Immune-responsive gene 1 protein links metabolism to immunity by catalyzing itaconic acid production. Proc. Natl. Acad. Sci. USA.

[10] Lampropoulou V., Sergushichev A., Bambouskova M., Nair S., Vincent E.E., Loginicheva E., Cervantes-Barragan L., Ma X., Huang S.C., Griss T. (2016). Itaconate Links Inhibition of Succinate Dehydrogenase with Macrophage Metabolic Remodeling and Regulation of Inflammation. Cell Metab..

[11] Cordes T., Wallace M., Michelucci A., Divakaruni A.S., Sapcariu S.C., Sousa C., Koseki H., Cabrales P., Murphy A.N., Hiller K. (2016). Immunoresponsive Gene 1 and Itaconate Inhibit Succinate Dehydrogenase to Modulate Intracellular Succinate Levels. J. Biol. Chem..

[12] Yu Z., Li X., Quan Y., Chen J., Liu J., Zheng N., Liu S., Wang Y., Liu W., Qiu C. (2024). Itaconate alleviates diet-induced obesity via activation of brown adipocyte thermogenesis. Cell Rep..

[13] Park A., Kim K.E., Park I., Lee S.H., Park K.Y., Jung M., Li X., Sleiman M.B., Lee S.J., Kim D.S. (2023). Mitochondrial matrix protein LETMD1 maintains thermogenic capacity of brown adipose tissue in male mice. Nat. Commun..

[14] Fujiwara Y., Hizukuri Y., Yamashiro K., Makita N., Ohnishi K., Takeya M., Komohara Y., Hayashi Y. (2016). Guanylate-binding protein 5 is a marker of interferon-γ-induced classically activated macrophages. Clin. Transl. Immunol..

[15] Eom J., Kim J.J., Yoon S.G., Jeong H., Son S., Lee J.B., Yoo J., Seo H.J., Cho Y., Kim K.S. (2019). Intrinsic expression of viperin regulates thermogenesis in adipose tissues. Proc. Natl. Acad. Sci. USA.

[16] Nair S., Huynh J.P., Lampropoulou V., Loginicheva E., Esaulova E., Gounder A.P., Boon A.C.M., Schwarzkopf E.A., Bradstreet T.R., Edelson B.T. (2018). Irg1 expression in myeloid cells prevents immunopathology during M. tuberculosis infection. J. Exp. Med..

[17] Sugimoto S., Mena H.A., Sansbury B.E., Kobayashi S., Tsuji T., Wang C.H., Yin X., Huang T.L., Kusuyama J., Kodani S.D. (2022). Brown adipose tissue-derived MaR2 contributes to cold-induced resolution of inflammation. Nat. Metab..

[18] Yoshida Y., Shimizu I., Shimada A., Nakahara K., Yanagisawa S., Kubo M., Fukuda S., Ishii C., Yamamoto H., Ishikawa T. (2022). Brown adipose tissue dysfunction promotes heart failure via a trimethylamine N-oxide-dependent mechanism. Sci. Rep..

[19] Crossley J.L., Ostashevskaya-Gohstand S., Comazzetto S., Hook J.S., Guo L., Vishlaghi N., Juan C., Xu L., Horswill A.R., Hoxhaj G. (2023). Itaconate-producing neutrophils regulate local and systemic inflammation following trauma. JCI Insight.

[20] Bambouskova M., Gorvel L., Lampropoulou V., Sergushichev A., Loginicheva E., Johnson K., Korenfeld D., Mathyer M.E., Kim H., Huang L.H. (2018). Electrophilic properties of itaconate and derivatives regulate the IκBζ-ATF3 inflammatory axis. Nature.

[21] Bi X., Wu X., Chen J., Li X., Lin Y., Yu Y., Fang X., Cheng X., Cai Z., Jin T. (2024). Characterization of ferroptosis-triggered pyroptotic signaling in heart failure. Signal Transduct. Target. Ther..

[22] Huang M., Zhang P.P., Tang Y.Y., Li M., Jiang J.M., Tang X.Q. (2025). Itaconate Attenuates Homocysteine-induced Nod-like Receptor Family Protein 3 Inflammasome-mediated Pyroptosis in Hippocampal Neurons: Involvement of Inhibiting Succinate Dehydrogenase Complex Subunit A Level. J. Physiol. Investig..

[23] Duan X., Hu M., Yang L., Zhang S., Wang B., Li T., Tan Y., Li Y., Liu X., Zhan Z. (2023). IRG1 prevents excessive inflammatory responses and cardiac dysfunction after myocardial injury. Biochem. Pharmacol..

[24] Blanco L.P., Patino-Martinez E., Nakabo S., Zhang M., Pedersen H.L., Wang X., Carmona-Rivera C., Claybaugh D., Yu Z.X., Desta E. (2022). Modulation of the Itaconate Pathway Attenuates Murine Lupus. Arthritis Rheumatol..

[25] Wang G.X., Zhao X.Y., Meng Z.X., Kern M., Dietrich A., Chen Z., Cozacov Z., Zhou D., Okunade A.L., Su X. (2014). The brown fat-enriched secreted factor Nrg4 preserves metabolic homeostasis through attenuation of hepatic lipogenesis. Nat. Med..

[26] Ding Y., Su J., Shan B., Fu X., Zheng G., Wang J., Wu L., Wang F., Chai X., Sun H. (2024). Brown adipose tissue-derivked FGF21 mediates the cardioprotection of dexmedetomidine in myocardial ischemia/reperfusion injury. Sci. Rep..

[27] Liu Y., Chen M. (2023). Neuregulin 4 as a novel adipokine in energy metabolism. Front. Physiol..

[28] Chen Z., Wang G.X., Ma S.L., Jung D.Y., Ha H., Altamimi T., Zhao X.Y., Guo L., Zhang P., Hu C.R. (2017). Nrg4 promotes fuel oxidation and a healthy adipokine profile to ameliorate diet-induced metabolic disorders. Mol. Metab..

