# Supplementary material for: Itaconate Promotes Cold Adaptation and Myocardial Protection by Enhancing Brown Adipose Tissue Metabolism
Source: Metabolites. 2026 Jan 12;16(1):66. doi: 10.3390/metabo16010066 (PMC12844064; doi:10.3390/metabo16010066)
Supplement: Supplementary file 1 [file metabolites-16-00066-s001.zip › metabolites-4005786-supplementary.pdf]

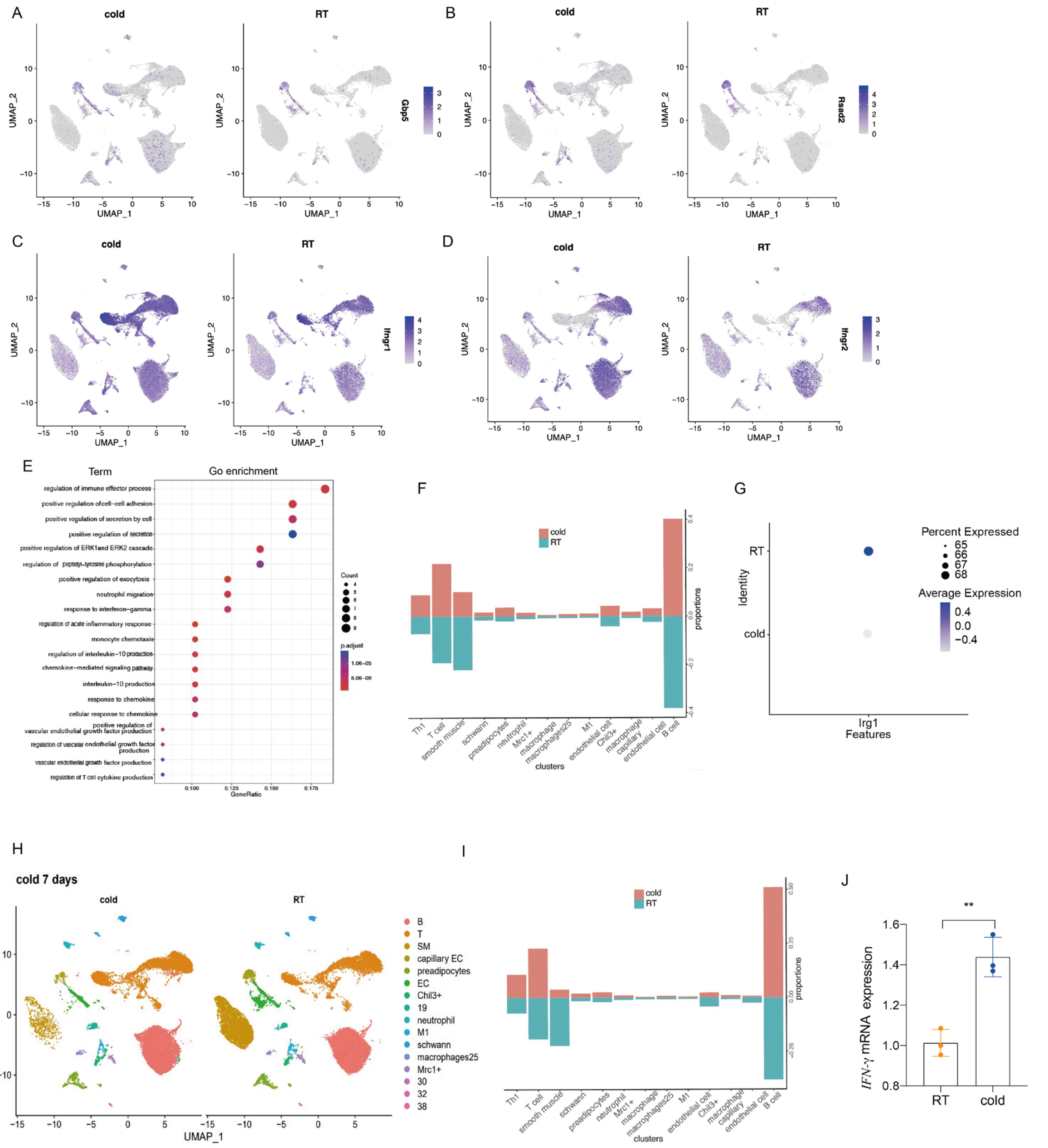

Supplemental Figure S1. Single-cell RNA sequencing analysis of cellular and transcriptional levels in iBAT SVF under cold stimulation and room temperature treatment. (A) FeaturePlots showing the expression of Gbp5 in BAT SVF cells after 2-day cold stimulation and under room temperature conditions. (B) FeaturePlots showing the expression of Rsad2 in BAT SVF cells after 2-day cold stimulation and under room temperature conditions. (C) FeaturePlots showing the expression of Ifngr1 in BAT SVF cells after 2-day cold stimulation and under room temperature conditions. (D) FeaturePlots showing the expression of Ifngr2 in BAT SVF cells after 2-day cold stimulation and under room temperature conditions. (E) Gene Ontology (GO) analysis of highly expressed genes in macrophage25 after 2-day cold stimulation. (F) Statistics of cell type proportions in BAT SVF after 2-day cold stimulation and under room temperature conditions. (G) The dot plot showing a significant decrease in Irg1 expression in neutrophils after 2-day cold stimulation. (H) t-distributed stochastic neighbor embedding (tSNE) plot of iBAT SVF from 7-day cold-stimulated and room temperature control groups. (I) Statistics of cell type proportions in BAT SVF after 7-day cold stimulation and under room temperature conditions. (J) The transcriptional level of IFN-γ in iBAT adipose tissue of mice under cold stimulation for 7 days and room temperature conditions was detected by RT-qPCR. Statistical significance was determined by 2-tailed Student's t-test. (**$p<0.01$, ns: non-significant).

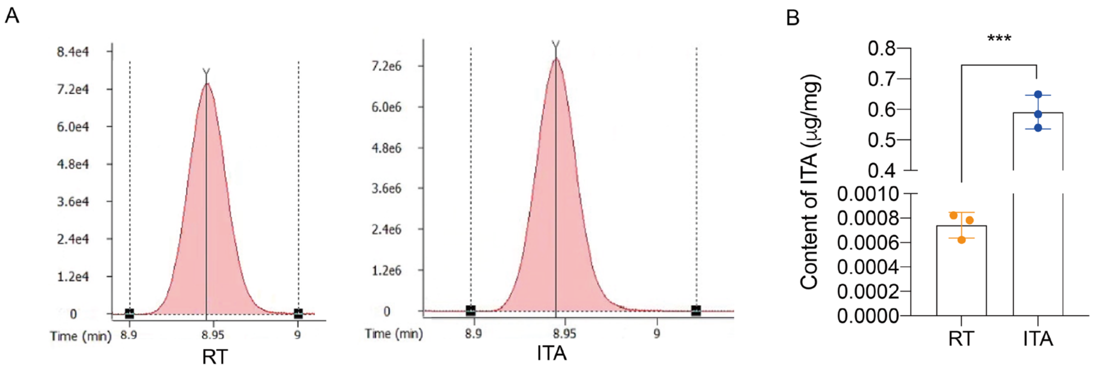

Supplemental Figure S2. Analysis of ITA Content in BAT after Intragastric Administration of ITA. (A) Chromatographic peaks of ITA in BAT from ITA-treated and control groups. (B) ITA content in BAT from ITA-treated and control groups. (***$p<0.001$).
